# Effects of the enhanced recovery after surgery (ERAS) protocol on the postoperative stress state and short‐term complications in elderly patients with colorectal cancer

**DOI:** 10.1002/cnr2.1979

**Published:** 2024-02-13

**Authors:** He Han, Rong Wan, Jixiang Chen, Xin Fan, LiWen Zhang

**Affiliations:** ^1^ Department of Gastrointestinal surgery Affiliated Hospital of Jiangsu University Zhenjiang China

**Keywords:** colorectal cancer, elderly, enhanced recovery after surgery, short‐term complications, stress

## Abstract

**Objective:**

The aim of this study was to evaluate the feasibility and necessity of enhanced recovery after surgery in elderly patients with colorectal cancer by observing inflammatory markers and postoperative complications.

**Methods:**

Hospitalized colorectal cancer patients from the Affiliated Hospital of Jiangsu University from January 2021 to September 2022 were included in the study and divided into two groups: Enhanced Recovery After Surgery (ERAS) and non‐ERAS. Data on postoperative inflammatory markers and complications were also collected.

**Results:**

A total of 313 patients with colorectal cancer were included: 182 in the ERAS group and 131 in the non‐ERAS group. The patients in the ERAS group had significantly shorter days of postoperative hospitalization, urinary catheter and drainage tube withdrawal times, and recovery of bowel function (*P* < .05) than those of the non‐ERAS group. Moreover, the ERAS group had lower hospitalization expenses than those of the non‐ERAS group (*P* < .05). However, the procalcitonin and tumor necrosis factor (TNF)‐α levels in the ERAS group was significantly lower than those in the non‐ERAS group on postoperative days 1 and 3 (*P* < .05), and the interleukin (IL)‐6 and IL‐10 levels in the ERAS group were significantly lower than those in the non‐ERAS group on the 1st, 3rd, and 5th postoperative days (*P* < .05). The C‐reactive protein (CRP) and white blood cell (WBC) levels in the ERAS group were lower than those in the non‐ERAS group on postoperative days 3 and 5 (*P* < .05). However, the hemoglobin levels did not differ significantly (*P* > .05). The albumin levels did not differ significantly between the two groups before surgery (*P* > .05); however, the albumin level in the ERAS group was higher than that in the non‐ERAS group on postoperative days 3 and 5 (*P* < .05). The ERAS patients had lower albumin levels after surgery than those of the non‐ERAS patients (*P* < .05).

**Conclusion:**

ERAS leads to a series of perioperative optimization measures, thereby reducing the postoperative stress response in elderly patients with colorectal cancer and the occurrence of perioperative complications.

## 1 INTRODUCTION

According to the colorectal cancer statistics published in 2020, a total of 147 950 patients with colorectal cancer were newly diagnosed in the United States, with the third highest mortality rate among all cancer patients.^1^ In China, 55 477 new patients of colorectal cancer were expected to be diagnosed in the country in 2020, ranking second among all types of tumors.^2^ Meanwhile, according to the statistics published in Europe and America, among the 140 000 patients diagnosed with colorectal cancer in 2018, about 60% were older than 65 years,^3^ and The National Health Commission reported that the number of elderly people in China reached more than 200 million by the end of 2021, accounting for 14.2% of the total population. The intensification of population aging globally is expected to cause an increase in the need for operation of elderly patients with colorectal cancer.

Age is considered to be a risk factor for postoperative complications; thus, as compared to younger patients, elderly patients have more complications,^4^, ^5^, ^6^ and those with colorectal cancer are usually complicated with underlying diseases, such as hypertension, diabetes, and heart disease, which could lead to difficulties in postoperative recovery and higher postoperative complications. Enhanced Recovery After Surgery (ERAS) has been widely used in the diagnosis and treatment of diseases, including colorectal cancer,^7^ and therefore provides a basis for the postoperative management of elderly patients with colorectal cancer. The Chinese Clinical Practice Guidelines for Accelerated Rehabilitation Surgery (2021 Edition)^8^ regulates the management protocol of ERAS in gastric surgery, colorectal surgery, hepatobiliary surgery, pancreatoduodenectomy, and other types of surgeries, which provides a guarantee for the application of ERAS techniques in patients with colorectal cancer aged over 65 years. Research has shown that the ERAS protocol is safe and feasible for patients aged >70 years.^9^

Studies have shown that the application of ERAS management protocol in elderly patients with colorectal cancer can accelerate postoperative recovery, reduce postoperative complications, and improve the three‐year survival rates.^10^, ^11^ However, there have only been a few studies on the implementation of accelerated rehabilitation surgery, specifically, for elderly patients with colorectal patients, and more clinical studies are needed to prove the effects of ERAS on the postoperative stress state and short‐term complications in patients with colorectal cancer.

With the aim of reducing the occurrence of perioperative complications and improving the efficacy of colorectal cancer in elderly patients, the ERAS protocol was applied in this study. Consequently, the feasibility and necessity of the use of ERAS in patients with colorectal cancer aged >65 years was studied by observing postoperative stress indicators and complications.

## 2 STUDY DATA AND METHODS

### 2.1 General information of patients

Patients with colorectal cancer from January 2021 to September 2022 at the Affiliated Hospital of Jiangsu University were recruited and divided into two groups: the ERAS group and the non‐ERAS group, according to the perioperative management. There are two wards in the hospital, designating the two groups in separate wards. Then, patients who would be undergoing surgery are randomly selected from either ward. All patients in the ERAS group signed an informed consent form upon admission and could withdraw from the ERAS group at any time. Informed consent forms were also required from the patients in the non‐EARS group, and an approval for the research was obtained from the ethics committee of the hospital in compliance with the ethical guidelines outlined in the 1964 Declaration of Helsinki (Grant No:KY2022H1014‐02).

### 2.2 Inclusion and exclusion criteria for the enrolled patients

The patients' inclusion criteria were as follows: (1) age ≥65 years; (2) no chemotherapy or other neoadjuvant chemotherapy before surgery; (3) consent to receive radical surgery (total mesorectal excision [TME] or complete mesocolic excision [CME]); and (4) signed consent form to receive ERAS from the patients or their family members.

The patients' exclusion criteria were as follows: (1) patients with severe organ dysfunction or intolerance to surgery; (2) patients with complete intestinal obstruction or receiving an intestinal stent; (3) patients requiring palliative surgery or emergency surgery; (4) patients requiring combined resection of surrounding organs; (5) patients who did not follow the ERAS treatment protocols or whose data did not meet the requirements; and (6) patients who did not agree to participate in the ERAS treatment.

### 2.3 Perioperative management

Based on multiple versions of the Chinese Clinical Diagnosis and Treatment Standards for the ERAS protocol and in consideration of the actual conditions of the patients and resources, a characteristic ERAS management protocol has been established for the patients in the study (Table 1). Patients in the ERAS group were strictly managed according to safety requirements, whereas those in the non‐ERAS group were managed according to traditional concepts. Then, the physician included in the study determined the number of days it took for urinary tube extraction and drainage tube extraction as well as the hospitalization duration of the patients.

**TABLE 1:** Specific perioperative management of the enhanced recovery after surgery (ERAS) and non‐eras groups.

ERAS group	Non‐ERAS group
**Preoperative**	
Strengthening of the publicity of ERAS and patient education was done to obtain their cooperation as well as that of their families. A fatigue monitor and anxiety scale was used to evaluate preoperative anxiety and fatigue.	Regular publicity and patient education were done. No fatigue monitor and anxiety scale were used.
Evaluation of preoperative nutrition and deep vein thrombosis were done.	Evaluation of preoperative nutrition and deep vein thrombosis were done.
Gastric emptying was evaluated the day before surgery.	Gastric emptying was not evaluated.
Non‐routine fasting and water prohibition were done before surgery. Food intake was restricted the night before the surgery at 22:00, while fluid intake was restricted 2 h before surgery to meet the anesthetic requirements. To avoid hunger stress and postoperative insulin resistance, 400 mL and 400 mL of dried suji and maltodextrin fructose drink, a high energy carbohydrate, were taken orally the night before and 2 h before the operation, respectively.	Fasting was observed for 12 h, and water was prohibited 6 h before the surgery.
Abdominal transversal fascia block was performed by the anesthesiologist half an hour before the operation.	Abdominal transversal fascia block was not done.
Compound polyethylene glycol electrolyte powder (139.12 g) was taken orally before the surgery.	Same as that in the ERAS group.
Prophylactic cefuroxime (1.5 g) was used 30 min before the surgery.	Same as that in the ERAS group.
**Intraoperative**	
General endotracheal anesthesia and epidural block anesthesia were administered.	General endotracheal anesthesia was administered.
Anal temperature was monitored using a deep body temperature probe. Air insulation blanket was used during the operation to prevent stress caused by low temperature and its effect on coagulation function.	The room temperature was maintained at 24–26 °C using an air insulation blanket during the operation. Anal temperature was not monitored.
Intraoperative blood glucose monitoring was done.	Intraoperative blood glucose monitoring was not done.
Focus was given on controlling the rate of fluid replenishment and monitoring the urine volume.	Same as that in the ERAS group.
**Postoperative**	
Cox‐II receptor blockers were administered orally and intravenously for analgesia and were added according to the patients' postoperative pain scores and follow‐up results to avoid or reduce the use of opioid analgesics, which could reduce the impact on the recovery of intestinal peristalsis function.	Postoperative pump analgesia was administered.
Food and fluid intake were resumed as soon as possible, and less than 25 mL/h of water was given after reversal of the effects of anesthesia. The patient was instructed to continue to take dried vegetables and other fluids orally from the first day after surgery.	Routine fasting was done, and the patient resumed intake of small amounts of fluid after reversal of the effects of anesthesia.
A detailed activity schedule was provided, and the patients were encouraged to get out of bed and walk as soon as possible, under the premise of sufficient pain relief, so as to facilitate the recovery of intestinal function. Four to six trips were done on the first day, and six to eight trips were done on the second day, recording and the daily distance covered.	Patient education was given; however, a detailed activity schedule was not provided.
The catheter was removed on the first day after surgery to reduce patient discomfort.	The catheter was removed once the patient felt the urge to urinate.
If a drainage tube was placed, this was removed as soon as possible.	If a drainage tube was placed, this was pulled out before discharge.

### 2.4 Observation indicators

According to the inclusion and exclusion criteria, we identified and selected the participants who could be enrolled in this study, and their general clinical data were collected: age, sex, body mass index (BMI), nutrition risk screening score (NRS)‐2002 score, presence of comorbidities such as hypertension or diabetes, tumor size, number of lymph node dissection, TNM stage, rate of laparoscopic surgery, and presence of fistula. The clinical staging of patients was based on the American Joint Committee on Cancer (AJCC) eighth edition grading system. Data on postoperative recovery was also collected: postoperative hospitalization days, urinary tube extraction time, drainage tube extraction time, recovery of bowel function, hospitalization cost, cost of Western medicine hospitalization, and frequency of readmission 30 d after surgery. The stress indices of the patients before surgery and on postoperative days 1, 3, 5, and 7 were analyzed, namely: the levels of procalcitonin, C‐reactive protein (CRP), tumor necrosis factor‐alpha (TNF‐α), interleukin (IL)‐6, IL‐10, albumin, hemoglobin (HB), and white blood cell (WBC) count; the postoperative complications of patients and postoperative electrolyte abnormalities such as hypokalemia, hyponatremia, or hypochloremia; and the number of patients admitted to the intensive care unit (ICU).

### 2.5 Data processing and analysis

In this study, statistical analysis was conducted using the SPSS 25.0 software. The measurement data were presented as mean ± standard deviation (X ± s). To assess the differences between groups, an independent sample t‐test was performed. On the other hand, the statistical data were expressed as a percentage (%), and comparisons between groups were made using either the chi‐square test or Fisher's exact probability method. Statistical significance was set at *P* < .05.

## 3 RESULTS

### 3.1 Comparison of the patients' general clinical data

This study included 313 patients diagnosed with colorectal cancer, with 182 and 131 participants assigned to the ERAS and non‐ERAS groups, respectively. No significant differences were observed between the two groups in terms of age, sex, BMI, NRS‐2002 score, presence of hypertension or diabetes, rate of laparoscopic surgery, or other basic physical conditions (*P* > .05; Table 2). Moreover, in terms of the tumor size, number of lymph node dissections, clinical stage of the tumor, or the use of prophylactic stoma, there were no notable differences between the two groups (*P* > .05; Table 2).

**TABLE 2:** General clinical data of patients included in the study.

Determinant	ERAS group (*n* = 182)	Non‐ERAS group (*n* = 131)	t/χ2‐value	*P*‐value
Gender [example (%)]	182	131	0.130	.718
Male	101 (55.49)	70 (53.44)		
Female	81 (44.51)	61 (46.56)		
Age (years,^**−**^X ± s)	72.62 ± 5.70	73.61 ± 6.37	0.015	.988
BMI (kg/m^2^, ^**−**^X ± s)	23.05 ± 3.48	23.79 ± 3.46	1.860	.064
NRS 2002 score [example (%)]			1.930	.587
2 points	66 (36.3)	46 (35.1)		
3 points	70 (38.5)	59 (45.1)		
4 points	32 (17.6)	17 (13.0)		
5 points	14 (7.6)	9 (6.8)		
Hypertension [example (%)]			0.078	.938
Yes	102 (56.0)	74 (56.5)		
No	80 (44.0)	57 (43.5)		
Diabetes mellitus			0.804	.422
Yes	31 (17.0)	27 (20.6)		
No	151 (83.0)	104 (79.4)		
Tumor size (cm,^**−**^X ± s)	4.64 ± 2.67	4.39 ± 1.83	0.926	.355
Number of lymph nodes dissected (^**−**^X ± s)	12.70 ± 6.77	11.86 ± 4.87	1.212	.226
TNM staging [example (%)]			6.563	.161
0	13 (7.1)	7 (5.3)		
I	29 (15.9)	34 (26.0)		
II	56 (30.8)	43 (32.8)		
III	73 (40.1)	39 (29.8)		
IV	11 (6.1)	8 (6.1)		
Prophylactic stoma [cases (%)]			0.024	.981
Yes	21 (11.5)	15 (11.4)		
No	161 (88.5)	116 (88.6)		
Laparoscopic surgery			0.7832	.3762
Yes	175 (96.7)	124 (94.7)		
No	6 (3.3)	7 (5.3)		

Abbreviations: BMI, Body mass index; NRS, Nutrition risk scale.

### 3.2 Postoperative recovery of the patient

As compared to those of the patients in the non‐ERAS group, the patients in the ERAS group had significantly shorter postoperative hospitalization days, urinary tube extraction time, drainage tube extraction time, and anal recovery of bowel function (*P* < .05; Table 3). The patients in the ERAS group had hospitalization costs as compared to that of the patients that in the non‐ERAS group (*P* < .05; Table 3); however, there was no significant difference in drug costs during hospitalization between the two groups (*P* > .05; Table 3). Additionally, electrolyte abnormalities and the rate of readmission after discharge were not significantly different between the two groups (*P* > .05; Table 3). We calculated the cost‐effectiveness of the 30‐d postoperative readmission rate based using the method proposed by Jing et al.^12^ The cost effectiveness of the ERAS group was 53 901, while that of the non‐ERAS group was 60 364. Thus, the ERAS group was superior to the non‐ERAS group in this regard.

**TABLE 3:** Postoperative recovery of patients in the study.

	ERAS group (*n* = 182)	Non‐ERAS group (*n* = 131)	t/ χ2 –value	*P*‐value
Postoperative hospitalization days (days,^**−**^X ± s)	10.67 ± 2.88	12.45 ± 3.31	5.065	*P* < .001
Catheter extraction time (days,^**−**^X ± s)	3.51 ± 2.01	7.58 ± 2.46	16.08	*P* < .001
Drainage tube extraction time (days,^**−**^X ± s)	9.18 ± 2.82	10.77 ± 3.51	4.438	*P* < .001
Recovery of bowel function (days,^−^X ± s)	2.40 ± 0.91	3.00 ± 1.00	5.520	*P* < .001
Hospitalization cost (yuan,^**−**^X ± s)	53 012 ± 10 147	59 440 ± 12 164	5.084	*P* < .001
Drug cost (yuan,^**−**^X ± s)	11 868 ± 6319	12 711 ± 6314	1.165	.2450
Electrolyte abnormality [cases (%)]			1.104	.2934
YES	76 (41.8)	47 (35.9)		
NO	106 (58.2)	84 (64.1)		
Readmission [cases (%)]				
YES	3 (1.6)	2 (1.5)	–	1.00
NO	179 (98.4)	129 (98.5)		

### 3.3 Stress indices and albumin levels of patients before and after surgery

Based on the analysis of the preoperative levels of procalcitonin, CRP, TNF‐α, IL‐6, IL‐10, HB, and WBC, a statistically significant divergence was found between the two groups (*P* < .05; *P* > .05). However, the procalcitonin and TNF‐α levels in the ERAS group were significantly lower than those in the non‐ERAS group on postoperative days 1 and 3 (*P* < .05), and the IL‐6 and IL‐10 levels in the ERAS group were significantly lower than those in the non‐ERAS group on postoperative days 1, 3, and 5 (*P* < .05). The CRP level and WBC count on postoperative days 3 and 5 in the ERAS group had significantly lower reductions compared to those in the non‐ERAS group (*P* < .05). However, the postoperative HB levels in the two groups were not significantly different (*P* > .05). In terms of the albumin level, there was no significant difference between the two groups before surgery (*P* > .05); however, the albumin level in the ERAS group was significantly higher than that in the non‐ERAS group on postoperative days 3 and 5 (*P* < .05; Table 4).

**TABLE 4:** Stress indices and albumin levels of patients before and after surgery.

Determinant	ERAS group (*n* = 182)	Non‐ERAS group (*n* = 131)	*t*‐value	*P*‐value
Procalcitonin (μg/L, ^**−**^X ± s)					
1 day before surgery	0.124 ± 0.13	0.105 ± 0.08	1.4830	.1392	
Postoperative day 1	1.980 ± 3.48	4.540 ± 3.82	6.1620	*P* < .001	
3 days after surgery	0.430 ± 0.42	0.896 ± 0.79	6.7450	*P* < .001	
5 days after surgery	0.309 ± 0.26	0.343 ± 0.66	0.6306	.5288	
7 days after surgery	0.174 ± 0.20	0.206 ± 0.23	1.3930	.1647	
CRP (mg/L, ^**−**^X ± s)					
1 day before surgery	3.68 ± 10.10	3.82 ± 8.04	0.1315	.8955	
Postoperative day 1	50.38 ± 33.72	52.08 ± 40.98	0.4018	.6881	
3 days after surgery	49.39 ± 41.46	69.35 ± 41.87	4.1840	*P* < .001	
5 days after surgery	25.67 ± 24.64	49.89 ± 29.38	7.9200	*P* < .001	
7 days after surgery	19.82 ± 22.45	19.22 ± 30.79	0.1994	.8421	
TNF‐α (pg/mL, ^**−**^X ± s)					
1 day before surgery	13.93 ± 4.27	13.21 ± 3.58	1.573	.1168	
Postoperative day 1	59.53 ± 14.15	66.80 ± 17.86	4.044	*P* < .001	
3 days after surgery	39.89 ± 7.63	43.57 ± 10.08	3.676	.0003	
5 days after surgery	32.75 ± 6.81	34.01 ± 8.66	1.440	.1509	
7 days after surgery	26.03 ± 5.36	25.12 ± 7.50	1.252	.2115	
IL‐6 (pg/mL, ^**−**^X ± s)					
1 day before surgery	8.34 ± 13.06	7.22 ± 10.11	0.8203	.4127	
Postoperative day 1	62.97 ± 97.07	125.7 ± 124.6	5.003	*P* < .001	
3 days after surgery	18.94 ± 18.48	83.97 ± 75.67	11.50	*P* < .001	
5 days after surgery	16.05 ± 27.03	38.08 ± 35.67	6.215	*P* < .001	
7 days after surgery	13.52 ± 12.88	13.00 ± 15.91	0.3190	.7499	
IL‐10 (pg/mL, ^**−**^X ± s)					
1 day before surgery	6.97 ± 6.60	5.97 ± 5.02	1.457	.1461	
Postoperative day 1	27.27 ± 15.86	36.75 ± 16.97	5.066	*P* < .001	
3 days after surgery	17.08 ± 10.50	25.46 ± 14.69	5.887	*P* < .001	
5 days after surgery	10.88 ± 6.90	16.48 ± 10.95	5.540	*P* < .001	
7 days after surgery HB (g/L, ^**−**^X ± s)	5.91 ± 3.75	6.78 ± ±.36	1.690	.0921	
1 day before surgery	118.0 ± 21.64	117.7 ± 20.02	0.1248	.9008	
Postoperative day 1	114.3 ± 21.76	114.5 ± 15.70	0.0897	.9286	
3 days after surgery	112.9 ± 19.82	111.5 ± 18.41	0.6350	.5259	
5 days after surgery	114.9 ± 19.69	115.3 ± 18.82	0.1806	.8568	
7 days after surgery	115.1 ± 17.51	118.8 ± 18.49	1.801	.0726	
The WBC (x 10 ^^ 9^, ^**−**^X ± s)					
1 day before surgery	6.14 ± 1.72	5.98 ± 1.82	0.7923	.4288	
Postoperative day 1	10.89 ± 3.26	10.64 ± 3.16	0.6779	.4983	
3 days after surgery	7.60 ± 2.47	9.00 ± 3.21	4.359	*P* < .001	
5 days after surgery	6.35 ± 2.11	7.60 ± 2.98	4.345	*P* < .001	
7 days after surgery	6.29 ± 2.65	6.75 ± 2.87	1.463	.1445	
Albumin (g/L, ^**−**^X ± s)					
1 day before surgery	38.10 ± 3.31	37.84 ± 3.06	0.7074	.4799	
Postoperative day 1	33.38 ± 3.45	33.72 ± 3.36	0.8695	.3852	
3 days after surgery	33.92 ± 3.28	32.13 ± 3.49	4.637	*P* < .001	
5 days after surgery	34.77 ± 2.98	32.73 ± 3.73	5.372	*P* < .001	
7 days after surgery	34.86 ± 3.47	34.61 ± 3.47	0.6288	.5299	

Abbreviations: CRP, C‐reactive protein; HB, Hemoglobin; IL‐10, Interleukin‐10; IL‐6, Interleukin‐6; TNF‐α, Tumor necrosis factor.

### 3.4 Postoperative complications of the patients

The postoperative complications included pulmonary infection, postoperative gastroparesis, incision infection, hypoproteinemia, anastomotic fistula/abdominal infection, and metabolic encephalopathy. A total of 10 patients in the ERAS group and nine patients in the non‐ERAS group had postoperative complications, displaying a statistically significant divergence (*P* < .05), and there was no significant difference in the probability of patients being admitted to the ICU between the two groups (Table 5).

**TABLE 5:** Postoperative complications of patients included in the study.

Determinant	ERAS group (*n* = 182)	Non‐ERAS group (*n* = 131)	χ2 –value	*P*‐value
Postoperative complication			14.07	*P* < .001
Yes	22 (12.1)	38 (29.0)		
No	160 (87.9)	93 (71.0)		
Pulmonary infection	5	8		
Postoperative gastroparesis	3	1		
Incision infection	4	7		
Hypoproteinemia	6	13		
Anastomotic Fistula/abdominal infection	3	5		
Metabolic encephalopathy	1	4		
Admitted in the ICU				>.9999
Yes	4 (2.2)	3 (2.3)		
No	178 (97.8)	128 (97.7)		

Abbreviation: ICU, Intensive care unit.

## 4 DISCUSSION

The concept of ERAS has been widely used in stomach cancer,^13^ colorectal cancer,^14^ liver transplantation,^15^ lung cancer,^16^ and many other diseases and has been shown to improve postoperative recovery and reduce postoperative complications. Hallam et al. demonstrated the feasibility of ERAS in elderly patients undergoing colorectal cancer surgery.^17^ However, owing to the characteristics of elderly patients coupled with the operation itself, the prognosis is not satisfactory in elderly patients with colorectal cancer, and ERAS in elderly patients is constantly developing and improving. A total of 313 patients were included in this study to analyze the effects of ERAS on postoperative stress and short‐term complications in elderly patients with colorectal cancer.

Surgical resection is the most common treatment method used for colorectal cancer, but it also leads to a large increase in the number of inflammatory cells and an increased stress response,^18^, ^19^ which are the main factors affecting the prognosis of patients.^20^ CRP, procalcitonin, TNF‐α, IL‐6, IL‐10, and WBC have been used as the most common markers of inflammatory stress,^21^, ^22^, ^23^ and Chen used these indices to compare the efficacy of laparoscopic surgery and conventional surgery in the treatment of colorectal cancer.^24^ In Wang's research, patients in the ERAS group had lower serum levels of TNF‐αIL‐6 and CRP as compared to those of the patients in the non‐ERAS group.^25^ By analyzing postoperative indices such as CRP levels, Zhao believed that ERAS could effectively and safely inhibit the postoperative stress response in patients with gastric cancer.^26^ In our study, the CRP, procalcitonin, TNF‐α, IL‐6, IL‐10, and WBC levels were statistically analyzed before and after surgery. On postoperative days 1, 3, and 5, the patients in the ERAS group had significantly lower levels of inflammatory markers than those of the patients in the non‐ERAS group, indicating that patients in the ERAS group were under a lower level of inflammatory stress, which is supported by Zhang's research.^27^

HB level is an independent risk factor for the overall survival in patients with cancer^28^ and can also reflect the nutritional status of patients. In our study, there were no significant differences in the HB levels between the two groups. As one of the most commonly used indicators in clinical work to assess the nutritional status of patients, many scholars have proven that a decrease in albumin level is closely related to poor prognosis^29^, ^30^ and can be used as a prognostic indicator. Studies have found that some inflammatory factors may also cause hypoproteinemia in patients^31^ and can be used as indicators of the inflammatory status of patients with tumors.^32^ In this study, the albumin levels of patients on postoperative days 3 and 5 were significantly lower in the ERAS group than those in the non‐ERAS group, which was consistent with the changes in common inflammatory factors, such as CRP, procalcitonin, and WBC, indicating that the use of the ERAS protocol can reduce the postoperative stress response in patients with colorectal cancer.

Elderly patients with colorectal cancer usually have underlying diseases, poor nutritional status, decreased physiological reserves and functional capacity,^33^ and decreased lung elasticity and thoracic compliance,^34^, ^35^ resulting in difficult perioperative recovery and higher occurrence of postoperative complications. In our study, postoperative hospitalization days, drainage tube extraction time, and urine tube extraction time in the ERAS group were better than those in the non‐ERAS group, which were improved through several perioperative optimization methods. Ni et al. demonstrated that the ERAS protocol could promote the recovery of bowel function and reduce postoperative complications in 1298 patients in a meta‐analysis,^36^ which is also consistent with the postoperative complications we observed. These studies demonstrate how the ERAS protocol could improve patients' hospitalization satisfaction and further improve their acceptance of the use of the ERAS protocol. The results indicate the feasibility and necessity of the ERAS protocol for elderly patients with colorectal cancer. The patients in the ERAS group had faster postoperative recovery, shorter duration of hospital stays, lower hospitalization costs, and less postoperative complications than those of the patients in the non‐ERAS group.

## 5 LIMITATIONS

However, this study has some shortcomings. This was a single‐center study and the sample size of this study was small, leading to limitations in the analysis and results and, consequently, reducing their credibility. Moreover, the long‐term patient outcomes were not tracked to further verify the long‐term efficacy of accelerated rehabilitation surgery. Of note, tumor location reflects the magnitude of surgical stress. Therefore, it is better to analyze the colon and rectum separately; however, due to sample size limitations, this was not done in this study.

## 6 CONCLUSION

In conclusion, our results demonstrated the feasibility and necessity of the ERAS protocol in elderly patients with colorectal cancer. This provides certain clinical evidence for the application of ERAS in elderly patients with colorectal cancer, leading to a series of perioperative optimization measures, which could be used to form a patient‐centered multidisciplinary cooperation model; thus, specifying detailed perioperative management plans according to individual differences in elderly colorectal cancer patients and reducing postoperative stress response and the occurrence of perioperative operative complications in elderly patients with colorectal cancer.

## AUTHOR CONTRIBUTIONS

**He Han:** Conceptualization (equal); data curation (equal); methodology (lead); resources (equal); writing – original draft (equal); writing – review and editing (equal). **Rong Wan:** Data curation (equal); methodology (equal); software (lead). **Jixiang Chen:** Formal analysis (equal); funding acquisition (equal); project administration (equal); resources (equal); supervision (equal). **Xin Fan:** Formal analysis (equal); funding acquisition (equal); project administration (equal); resources (equal); supervision (lead). **LiWen Zhang:** Conceptualization (equal); data curation (equal); formal analysis (equal); funding acquisition (equal); methodology (lead); resources (equal); writing – original draft (equal); writing – review and editing (equal).

## FUNDING INFORMATION

This study was sponsored by the Jinagsu Commission of Health (grant number: LKZ2023012) and the Social Development Project of Zhenjiang City (grant number: SH2022061).

## CONFLICT OF INTEREST STATEMENT

The authors declare that there are no conflicts of interest related to this study, including the authorship and/or publication of this article.

### ETHICS STATEMENT

This study was approved by the hospital's ethics committee in compliance with the ethical guidelines outlined in the 1964 Declaration of Helsinki (Grant No:KY2022H1014‐02).

## Data Availability

Data sharing is not applicable to this article as no new data were created or analyzed in this study.
